# Immunohistochemical Analysis of the Activation Status of the Akt/mTOR/pS6 Signaling Pathway in Oral Lichen Planus

**DOI:** 10.1155/2013/743456

**Published:** 2013-10-21

**Authors:** Georgios Prodromidis, Nikolaos G. Nikitakis, Alexandra Sklavounou

**Affiliations:** Department of Oral Pathology and Medicine, School of Dentistry, University of Athens, 2 Thivon Street, Goudi, 116 27 Athens, Greece

## Abstract

*Introduction*. Aberrations of the Akt/mTOR/pS6 pathway have been linked to various types of human cancer, including oral squamous cell carcinoma (OSCC). The purpose of this study was to evaluate the activation status of Akt, mTOR, and pS6 in oral lichen planus (OLP) in comparison with oral premalignant and malignant lesions and normal oral mucosa (NM). *Materials and Methods*. Immunohistochemistry for p-Akt, p-mTOR, and phospho-pS6 was performed in 40 OLP, 20 oral leukoplakias (OL), 10 OSCC, and 10 control samples of NM. *Results*. Nuclear p-Akt expression was detected in the vast majority of cases in all categories, being significantly higher in OL. Cytoplasmic p-Akt and p-mTOR staining was present only in a minority of OLP cases, being significantly lower compared to OL and OSCC. Phospho-pS6 showed cytoplasmic positivity in most OLP cases, which however was significantly lower compared to OL and OSCC. *Conclusions*. Overall, cytoplasmic p-Akt, p-mTOR, and phospho-pS6 levels appear to be significantly lower in OLP compared to OL and OSCC. However, the expression of these molecules in a subset of OLP cases suggests that activation of Akt/mTOR/pS6 may occur in the context of OLP, possibly contributing to the premalignant potential of individual cases.

## 1. Introduction

Oral lichen planus (OLP) is an immunologically mediated disease, the malignant potential of which has been a subject of intense investigation and ongoing controversy [1, 2]. Several published series of OLP with long-term follow-up have reported a variable rate of malignant transformation ranging from 0 to 12.5%, with most authorities suggesting that the actual percentage revolves around 1% [1, 2]. Although the overall malignant transformation rate appears low, the increased frequency of OLP in the general population makes it necessary to investigate the epidemiologic, clinical, and histopathologic factors related to the premalignant nature of OLP and also to elucidate the possible molecular mechanisms and pathways underlying oral carcinogenesis in the context of a preexisting OLP lesion [3].

In recent years, advances in molecular biology have allowed the dissection of several carcinogenesis-related signaling pathways and have offered insight into the molecular events and aberrations mediating cancer development and progression. Akt/mTOR/pS6 signaling has been identified as one of the most commonly implicated pathways in various types of human cancer, including oral squamous cell carcinoma [4, 5]. Akt is a serine-threonine kinase that functions as a downstream target and effector of phosphatidylinositol 3-kinase (PI3K). It is frequently activated in human cancers and precancerous lesions and is considered a key regulator of normal and cancerous cell growth and fate decisions [6, 7]. mTOR is one of the major targets of activated Akt, which in turn regulates a number of downstream molecules, such as ribosomal protein pS6, eventually controlling fundamental cell processes such as cell survival, proliferation, protein synthesis, and angiogenesis [4, 6, 7]. Dysregulations in upstream and downstream molecules of mTOR signaling appear to occur in 90–100% of HNSCC suggesting that markers and targets in the Akt/mTOR/pS6 pathway may be of particular clinical relevance [8].

The purpose of this study was to assess the immunohistochemical expression of the phosphorylated (activated) forms of Akt, mTOR, and pS6 in biopsy samples of OLP in comparison with oral leukoplakia (OL) with various degrees of dysplasia, oral squamous cell carcinoma (OSCC), and control cases of normal mucosa (NM), in order to evaluate the potential contribution of Akt/mTOR/pS6 signaling pathway aberrations in OLP malignant potential. 

## 2. Materials and Methods

### 2.1. Patients and Tumor Samples

Formalin-fixed, paraffin-embedded tissue samples from 40 patients diagnosed with OLP, 20 patients diagnosed with OL (including 5 hyperplasias, 5 mild dysplasias, 5 moderate dysplasias, and 5 severe dysplasias), and 10 patients diagnosed with OSCC at the Department of Oral Medicine and Pathology, University of Athens, Greece, between 2005 and 2008 were identified. Ten control cases of oral NM of healthy subjects were also included. Clinical records for these patients were reviewed, and information regarding the final diagnosis was obtained. Representative hematoxylin and eosin sections of each tumor were reviewed, and the diagnosis (as well as degree of dysplasia for OL and grade of differentiation for OSCC) was confirmed according to well-accepted diagnostic criteria [9].

### 2.2. Immunohistochemistry

Five micron-thick serial sections of formalin-fixed and paraffin-embedded tissues were immunostained in the Leica BOND-MAX fully automated immunohistochemistry system (Leica Biosystems Newcastle Ltd, Newcastle Upon Tyne, UK), by applying the NovoLink Polymer Detection System (Leica Biosystems Newcastle Ltd). For epitope retrieval, a high-temperature technique with citrate buffer was utilized. The sections were incubated in 3% hydrogen peroxide (Novocastra Peroxidase Block, Novocastra Leica Microsystems) to neutralize endogenous peroxidase activity; treated with Novocastra Protein Block to reduce nonspecific binding of primary antibody and polymer; incubated with primary antibodies; and treated with Novocastra Postprimary Block, containing 10% (v/v) animal serum in tris-buffered saline, to enhance penetration of the subsequent polymer reagent. Consequently, poly-HRP anti-mouse/rabbit IgG reagent (NovoLink Polymer) containing 10% (v/v) animal serum in tris-buffered saline was applied to localize the primary antibody, and the reaction product was visualized by incubation with the substrate/chromogen, 3,3′-diaminobenzidine (DAB) prepared from Novocastra DAB Chromogen and NovoLink DAB Substrate Buffer (Polymer), as a brown precipitate. Finally, the sections were counterstained with Novocastra Hematoxylin (0.02%).

The following primary antibodies were used: rabbit monoclonal antibody against phosphorylated Akt (1 : 100, p-Akt, phosphorylated at serine 473, Cell Signaling Technology Inc., no. 4060); rabbit monoclonal antibody against phosphorylated mTOR (1 : 100, p-mTOR, phosphorylated at serine 2448, Cell Signaling Technology Inc., no. 2976); and rabbit polyclonal antibody against phosphorylated S6 protein (1 : 100, phospho-pS6, phosphorylated at serine 235/236, Cell Signaling Technology Inc., no. 2211). Due to limited available tissue material, a few cases were not stained with all three antibodies. 

Appropriate positive control cases were used for both antibodies. As a negative control, sections were treated with phosphorylated buffered saline (PBS) with omission of the primary antibody.

The immunostains were reviewed by two independent evaluators (GP and NN). The interobserver variability was very low (<5% of cases). In cases in which there was initial disagreement, stains were reevaluated by the aforementioned investigators using a multiobserver microscope and discussed until consensus was reached.

Immunohistochemical reactivity for p-Akt, p-mTOR, and phospho-pS6 stains was graded in a semiquantitative manner according to the percentage of positive epithelial cells: (0) 0%, (1) <20%, (2) 20–50%, and (3) >50%, and the intensity of staining: (0) no staining, (1) weak, (2) moderate, or (3) strong, as compared to the negative control tissues. Moreover, a combined score of immunohistochemical positivity (0, 2–6) was calculated for each case by adding the individual scores for percentage of cells (0–3) and intensity of staining (0–3). In cases of OLP, OL, and NM, positive cells were identified within the whole epithelial thickness. For OSCC, immunostaining was evaluated in the tumor cell population. The subcellular localization of immunohistochemical staining (nuclear versus cytoplasmic staining pattern) was also recorded and, where appropriate, scored separately.

### 2.3. Statistical Analysis

Immunohistochemical scores (intensity, positivity, and total scores for p-Akt, p-mTOR, and phospho-pS6) were compared between OLP and the other categories (OL, OSCC, and NM) using nonparametric tests (Mann-Witney). A statistically significant difference was considered to be present at *P* ≤ 0.01 (following Bonferroni correction).

## 3. Results

### 3.1. p-Akt

**Figure 1:**
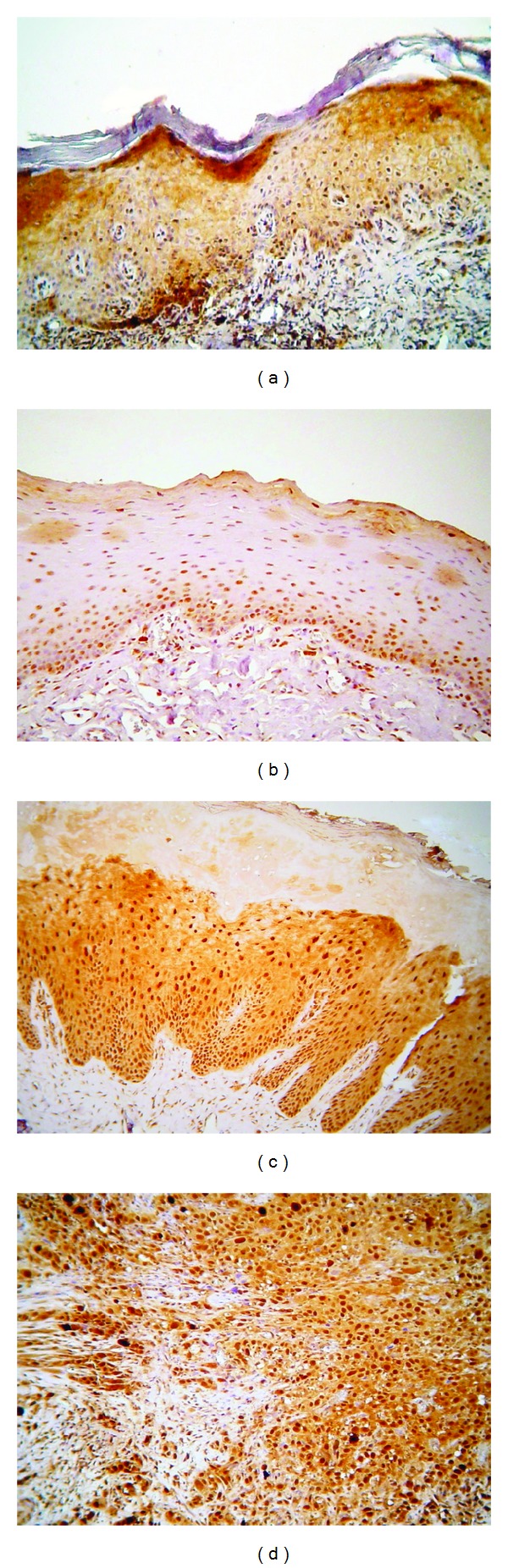
Immunohistochemical expression of phosphorylated Akt at serine 473 (p-Akt) in selected cases of (a) oral lichen planus (OLP), (b) normal mucosa (NM), (c) oral leukoplakia (OL), and (d) oral squamous cell carcinoma (OSCC) (immunohistochemistry, 100x magnifications).

p-Akt staining was noticed to be localized in the nucleus and/or the cytoplasm of the epithelial cells in the various cases studied (Figure 1). Because of the observed heterogeneity of p-Akt staining localization and taking into account previous studies reporting nuclear p-Akt staining in oral epithelial lesions, as well as neoplasms of diverse origin [10–15], both nuclear and cytoplasmic p-Akt immunoreactivities were analyzed.

#### 3.1.1. Nuclear p-Akt

Of 40 OLP cases studied for nuclear expression of p-Akt, 37 (92.5%) were positive, whereas 3 (7.5%) were negative. Of the positive cases, 7 (17.5%) showed nuclear p-Akt immunopositivity in <20% of epithelial cells, while 17 (42.5%) and 13 (32.5%) cases exhibited staining in 20–50% and >50% of epithelial cells, respectively; the average score for the percentage of positive epithelial cells for nuclear p-Akt was 2.00. On the other hand, the average score for the staining intensity was 1.53, corresponding to 15 (37.5%) cases that were stained weakly, 20 (50%) moderately, and 2 (5%) strongly. The average combined score of nuclear p-Akt immunohistochemical positivity in OLP was 3.53.

Regarding nuclear p-Akt staining in OL, all studied cases were positive. Based on the percentage of positive cells, 16 cases (94.1%) received score 3 and 1 case (5.9%) received score 2 for positivity (average score: 2.94). The average intensity score was 2.71; one case (5.9%) received score 1, 3 cases (17.6%) score 2, and 13 cases (76.5%) received score 3. The average combined score for nuclear p-Akt immunohistochemical positivity in oral leukoplakia was 5.65.

On the other hand, OSCC showed positivity in 70% of cases studied; the average positivity, intensity, and total scores were 1.8, 1.3, and 3.1, respectively. All NM cases were positive and the corresponding positivity, intensity, and total scores were 1.78, 1.78, and 3.56, respectively.

Statistical analysis revealed significant differences between OLP and OL regarding intensity (*P* < 0.0001), positivity (*P* < 0.0001), and total scores (*P* < 0.0001); in other words, OL received higher scores for p-Akt nuclear expression compared to OLP. In contrast, no significant differences between OLP and NM or between OLP and OSCC were noticed.

**Figure 2:**
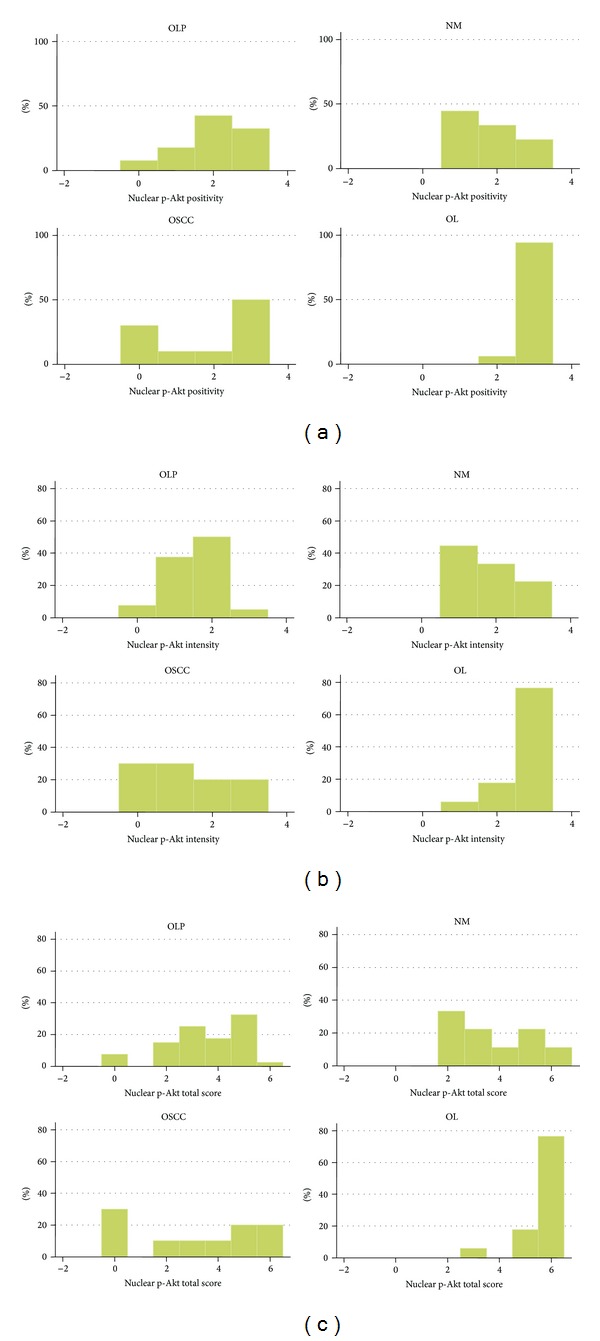
Graph of immunohistochemical results for nuclear p-Akt. Distribution of cases per lesion category according to (a) positivity score, (b) intensity score, and (c) total score. Abbreviations: OLP: oral lichen planus; NM: normal mucosa; OSCC: oral squamous cell carcinoma; OL: oral leukoplakia.

The results for nuclear p-Akt are summarized in Table 1 and Figure 2.

#### 3.1.2. Cytoplasmic p-Akt

Of the 40 OLP cases studied for cytoplasmic expression of p-Akt, 38 (95%) were negative and only 2 cases (5%) were positive. The two positive cases showed weak or moderate immunostaining in less than 20% of epithelial cells, so that the average positivity, intensity, and total scores for cytoplasmic p-Akt were 0.05, 0.08, and 0.05, respectively.

With regard to OL, 6 cases (35.3%) were negative and 11 cases (64.7%) were positive. The average positivity, intensity, and total scores for cytoplasmic p-Akt in OL were 0.94, 1.06, and 2.00, respectively. 

All NM cases were negative. In contrast, 70% of OSCC were positive; the average positivity, intensity, and total scores for cytoplasmic p-Akt in oral SCC were 1.10, 1.10, and 2.20, respectively.

Statistical analysis showed no significant differences between OLP and NM. However, the intensity, positivity, and total scores for p-Akt cytoplasmic expression were significantly lower in OLP compared to both OL (*P* < 0.0001) and OSCC (*P* < 0.0001).

**Figure 3:**
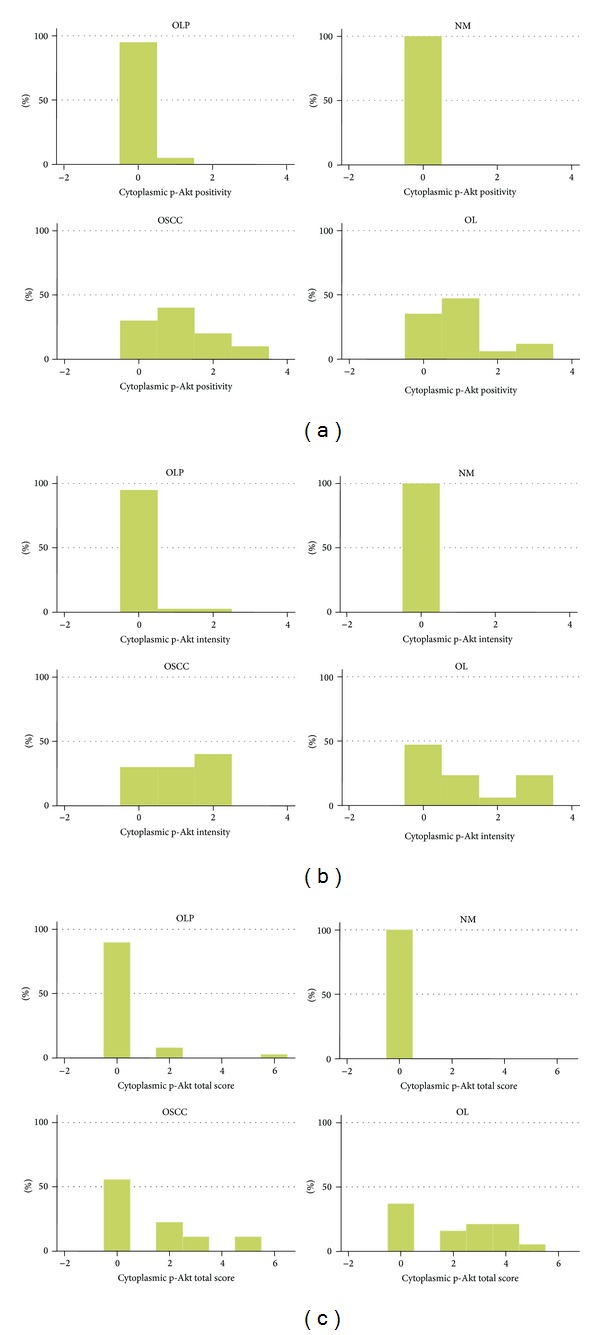
Graph of immunohistochemical results for cytoplasmic p-Akt. Distribution of cases per lesion category according to (a) positivity score, (b) intensity score, and (c) total score. Abbreviations: OLP: oral lichen planus; NM: normal mucosa; OSCC: oral squamous cell carcinoma; OL: oral leukoplakia.

The results for cytoplasmic p-Akt are summarized in Table 2 and Figure 3.

### 3.2. p-mTOR

**Figure 4:**
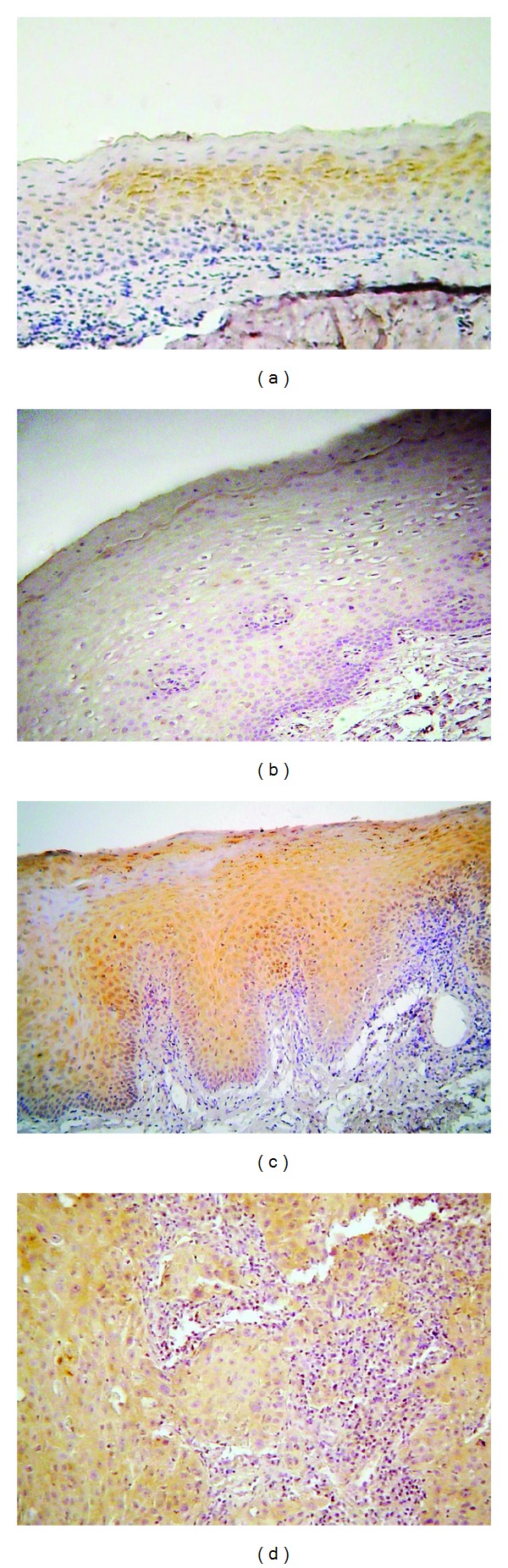
Immunohistochemical expression of phosphorylated mTOR (p-mTOR) in selected cases of (a) oral lichen planus (OLP), (b) normal mucosa (NM), (c) oral leukoplakia (OL), and (d) oral squamous cell carcinoma (OSCC) (immunohistochemistry, 100x magnifications).

p-mTOR was expressed in a subset of the cases studied showing exclusive cytoplasmic localization (with the exception of a single case of OLP showing nuclear expression) (Figure 4). 

Of the 39 OLP cases studied for immunohistochemical expression of p-mTOR, 35 (89.7%) were negative. Only 4 cases (10.3%) were positive, corresponding to 3 cases (7.7%) with immunoreactivity in <20% of epithelial cells and 1 case (2.6%) showing staining in >50% of epithelial cells. The average positivity, intensity, and total scores for p-mTOR in OLP were 0.154, 0.154, and 0.308, respectively. 

Regarding p-mTOR staining in OL, 7 cases (36.8%) were negative, and 12 cases (63.2%) were positive. More specifically, 5 cases (26.4%) received score 1 and 7 cases (36.8%) received score 2 for positivity (average score: 1.00). The average intensity score was 1.05, with 5 cases (26.4%) receiving score 1, 6 cases (31.6%) receiving score 2, and one case (5.3%) receiving score 3. The average total score of p-mTOR immunohistochemical positivity in OL was 2.05.

Of 9 studied OSCC cases, 5 (55.6%) were negative and 4 (44.4%) were positive. The average positivity, intensity, and total scores for p-mTOR in OSCC were 0.78, 0.56, and 1.33, respectively. In contrast, all NM cases were negative (average score 0). 

Statistical analysis showed that no significant differences existed between OLP and NM (despite the fact that all NM cases were negative as opposed to 10.3% of OLP cases being positive). On the other hand, the intensity, positivity, and total scores for p-mTOR immunohistochemical expression were significantly lower in OLP compared to both OL (*P* < 0.0001) and OSCC (*P* < 0.01).

**Figure 5:**
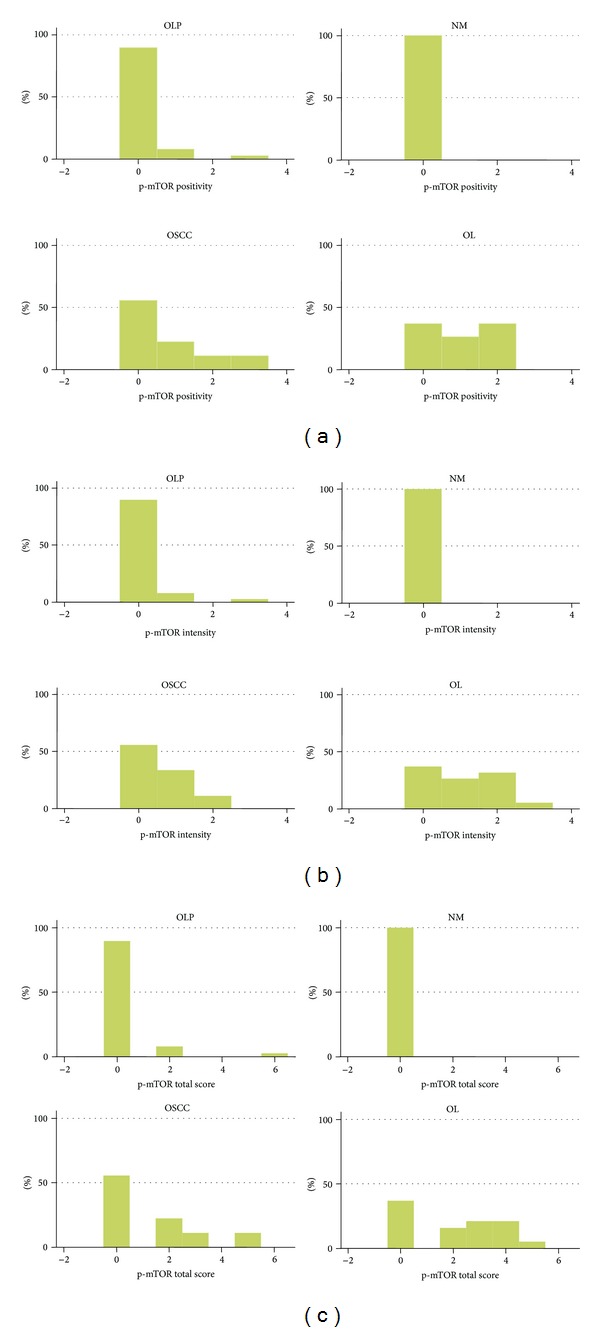
Graph of immunohistochemical results for p-mTOR. Distribution of cases per lesion category according to (a) positivity score, (b) intensity score, and (c) total score. Abbreviations: OLP: oral lichen planus; NM: normal mucosa; OSCC: oral squamous cell carcinoma; OL: Oral leukoplakia.

The results for p-mTOR are summarized in Table 3 and Figure 5.

### 3.3. Phospho-pS6

**Figure 6:**
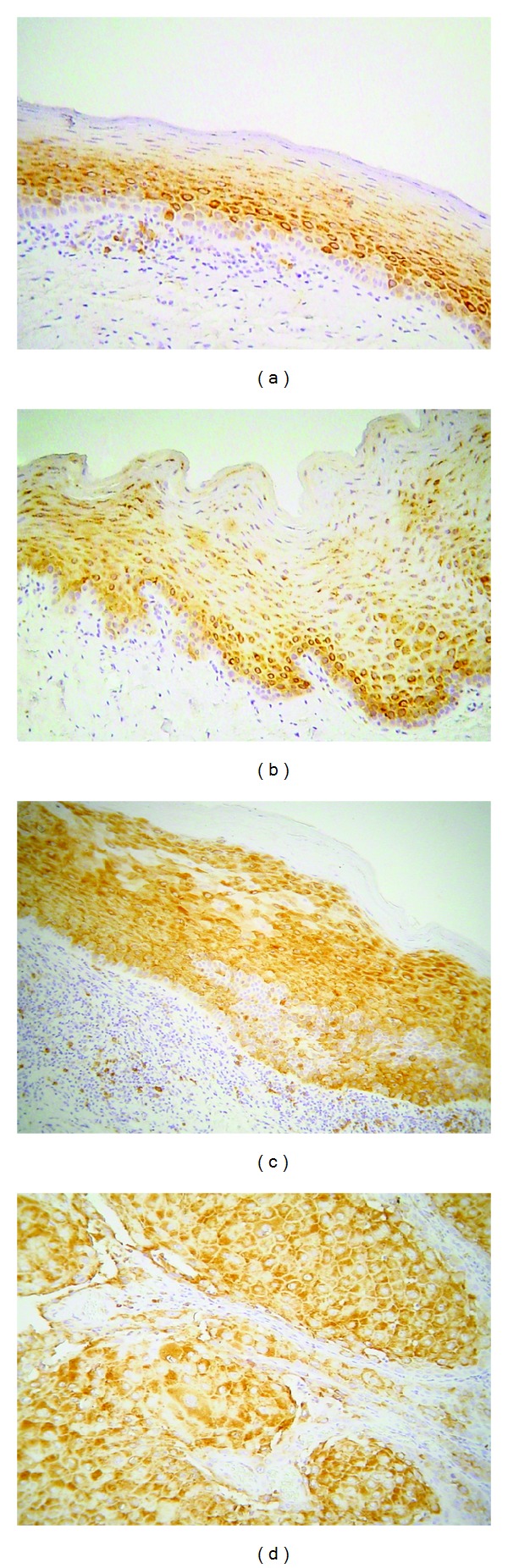
Immunohistochemical expression of phosphorylated ribosomal protein pS6 (phospho-pS6) in selected cases of (a) oral lichen planus (OLP), (b) normal mucosa (NM), (c) oral leukoplakia (OL), and (d) oral squamous cell carcinoma (OSCC) (immunohistochemistry, 100x magnifications).

Phosphorylated pS6 (phospho-pS6) was immunohistochemically detected in the majority of the cases studied; the staining pattern was cytoplasmic (Figure 6). 

Out of the 40 OLP cases, 36 (90%) were positive, while only 4 (10%) were negative. Most positive cases (24/40, 60%) showed immunopositivity in <20% of epithelial cells, while 9 (22.5%) and 3 (7.5%) showed staining in 20–50% and >50% of epithelial cells, respectively; the average score for percentage of positive epithelial cells for phospho-pS6 in OLP was 1.28. Regarding phospho-pS6 staining intensity, 21/40 (52.5%) cases were weak, while 15/40 (37.5%) were moderate; the average score for staining intensity was 1.28. Finally, the average combined score of phospho-pS6 immunostaining in OLP was 2.52.

All OL cases studied were positive for phospho-pS6 (16/16, 100%). Eight cases (50%) demonstrated immunoreactivity in 20–50% of epithelial cells, 2 cases (12.5%) were positive in <20% of cells, while 6 cases (37.5%) showed positivity in >50% of cells; the average positivity score was 2.25. On the other hand, the average intensity score was 1.81 corresponding to 8 cases (50%) receiving score 1, 3 cases (18.75%) receiving score 2, and 5 cases (31.25%) receiving score 3. The average total immunohistochemical score for phospho-pS6 in OL was 4.06.

Eight out of 9 OSCC cases (88.9%) were positive, most of them (7/9, 77.7%) showing strong immunoreactivity in >50% of tumors cells. The average positivity, intensity, and total scores for phospho-pS6 in OSCC were 2.33, 2.33, and 4.67, respectively. Finally, all NM cases were positive and the corresponding positivity, intensity, and total scores were 1.78, 1.78, and 3.56, respectively. 

Statistical analysis did not reveal significant differences in phospho-pS6 immunoreactivity between OLP and NM. However, the intensity, positivity, and total scores for phosphor-pS6 expression were significantly lower in OLP compared to both OL (*P* < 0.0004) and OSCC (*P* < 0.002).

**Figure 7:**
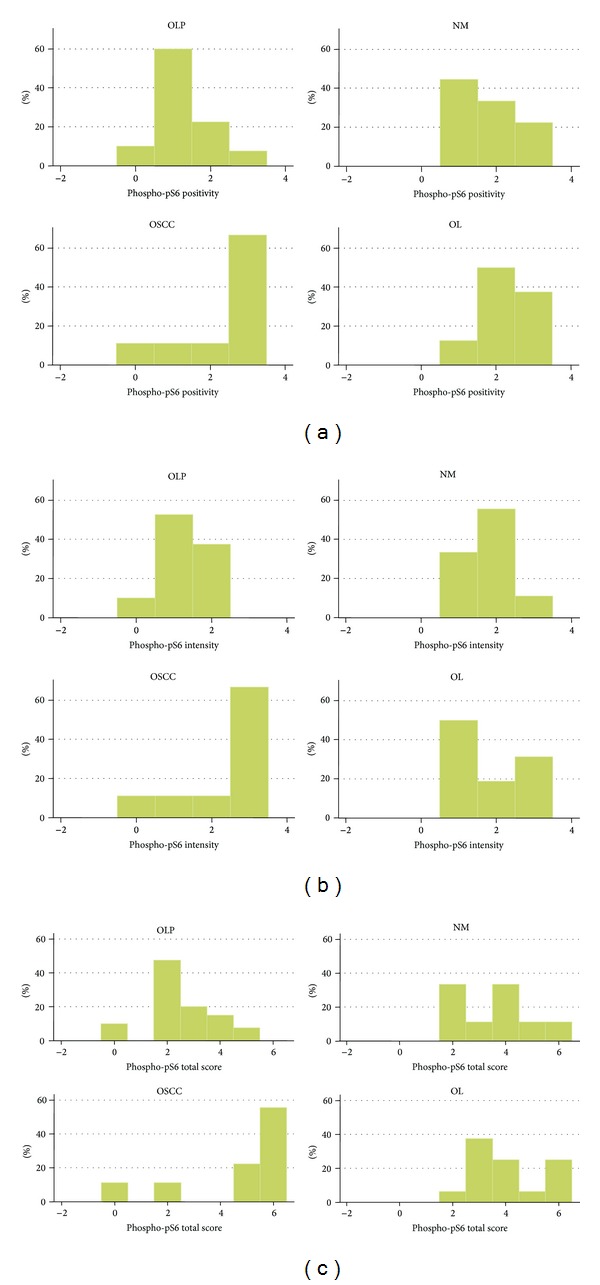
Graph of immunohistochemical results for phosphorylated ribosomal protein pS6 (phospho-pS6). Distribution of cases per lesion category according to (a) positivity score, (b) intensity score, and (c) total score. Abbreviations: oLP: Oral lichen planus; NM: normal mucosa; OSCC: oral squamous cell carcinoma; OL: oral leukoplakia.

The results for p-mTOR are summarized in Table 4 and Figure 7.

## 4. Discussion

The present study attempted to investigate the activation status of the Akt/mTOR/pS6 pathway in cases of OLP compared to cancerous (OSCC) and precancerous (OL) lesions and normal oral mucosa (NM) samples. Since phosphorylation of Akt, mTOR, and pS6 is necessary for their activation, the phosphorylated levels of these molecules were examined.

To evaluate Akt activation status, an antibody recognizing Akt phosphorylated at serine 473 was employed. It has been demonstrated that Akt activation involves interaction of its N-terminal pleckstrin homology (PH) domain with 3-phosphoinositides generated by the phosphoinositide 3-kinase (PI3K) with ensuing translocation of the molecule to the plasma membrane [12, 13]. Full Akt activation requires phosphorylation by PDK1 at Thr-308 and by PDK2 at Ser-473 [16, 17]. Since Akt phosphorylation at Ser-473 involves an mTOR- containing protein complex (mTORC2), it is also a marker of mTOR activity [12, 18]. On the other hand, PTEN (phosphatase and tensin homolog deleted on chromosome 10), a well-recognized tumor suppressor gene, is a negative regulator of Akt [19, 20]. Activated Akt dissociates from the plasma membrane and exerts its activity by phosphorylating both cytoplasmic and nuclear downstream effectors, including mTOR [15]. By doing so, Akt regulates a number of critical biological functions, such as cell growth and survival, apoptosis, and metabolism. In addition, Akt activation has been associated with cancer development and progression in various human cancers and has been proposed as a potential molecular marker of prognostic and therapeutic significance [4–6].

In the present study, both nuclear and cytoplasmic levels of p-Akt were evaluated. Although the mechanisms and the significance of this compartmentalization are largely unknown, previous investigations have shown variable distribution of p-Akt in different cellular compartments of oral premalignant and malignant lesions indicating that p-Akt localization may be related to differences in activity [10, 11]. In addition, differential subcellular (nuclear vs. cytoplasmic) p-Akt localization has been reported in other types of cancer and, as suggested, may play a significant role in determining its function [12–15]. We observed a high percentage of cases showing p-Akt nuclear positivity in the epithelial compartment in all categories studied, including NM and OLP. Only oral premalignant lesions (OL) demonstrated higher levels of nuclear p-Akt expression compared to OLP. On the other hand, cytoplasmic detection of p-Akt rendered different results: all NM cases were negative, whereas only 5% of OLP cases studied were positive. In contrast, a significant proportion of OL and OSCC cases (about 65% and 70%, resp.) were positive for cytoplasmic p-Akt. These results, taken together, suggest that cytoplasmic p-Akt may be a more meaningful indicator of the biological activity of the molecule in the context of oral carcinogenesis, which remains undetected under normal conditions and seems to be activated in the majority of OL and OSCC cases, as opposed to only a few OLP cases.

In the study of Pontes et al. [10], p-Akt was detected in all cases of NM, OL, and OSCC. Both severe dysplasia and OSCC cases showed higher p-Akt expression compared to NM; in addition, OSCC cases demonstrated higher levels of p-Akt immunostaining compared to dysplasias. On the other hand, no significant differences were observed among the three histological grades of oral dysplasia. Regarding the localization of immunostaining, Pontes et al. [10] reported that p-Akt was expressed mainly in both cytoplasmic and nuclear compartments, although restricted staining in either the nucleus or the cytoplasm was also occasionally observed. de Freitas Silva et al. [11] also evaluated p-Akt immunohistochemical levels in oral lesions and reported a significant increase in OL and OSCC compared to NM; no significant differences among OL cases with different degrees of dysplasia were noted. With regard to localization, de Freitas Silva et al. [11] reported that NM and OL cases showed p-Akt immunoreactivity limited to the nucleus, whereas OSCC cells expressed both nuclear and cytoplasmic immunostaining. The authors suggested that p-Akt may participate in the multistep process of oral carcinogenesis and may be associated with TWIST expression, a molecule involved in epithelial-mesenchymal transition [11]. It should be noted that the antibody used by Pontes et al. [10] and de Freitas Silva et al. [11] was specific for detecting p-Akt phosphorylation at threonine 308. In addition, analysis of the immunostaining was not performed separately in the nucleus and the cytoplasm of epithelial cells. Similar to our study, Wu et al. [21] analyzed the immunohistochemical expression of p-Akt in NM, OL, and OSCC, but not in OLP, using an antibody against Akt phosphorylated at serine 473. Interestingly, NM showed faint or weak staining, with an occasional lack of expression, which was predominantly located within the nucleus at the basal cell layer. Overall, there was a gradual increase in p-Akt immunostaining from NM to precancerous lesions and OSCCs.

Despite differences in methodology, our findings are in agreement with previous studies in that p-Akt was higher in oral precancerous and cancerous lesions compared to NM. Cytoplasmic p-Akt expression in a minority of OLP cases indicates that this molecule may not participate in the mechanisms underlying OLP pathogenesis. However, it could be hypothesized that certain OLP cases harbor abnormal Akt activity, which could be related to their potential for malignant transformation. In other words, OLP cases with cytoplasmic p-Akt immunostaining may share similar characteristics with OL and OSCC cases showing similar characteristics, thus theoretically being more suspicious for the accumulation of additional genetic and epigenetic alterations leading to cancer development. 

One major target of p-Akt is mTOR, which is activated through p-Akt-induced direct phosphorylation and inhibition of TSC2, a tumor suppressor protein that functions as a negative regulator of mTOR [22]. By controlling important downstream targets, mTOR exerts a crucial role in cell fate decisions, so that mTOR signaling dysregulations have been implicated in various forms of human cancer [4, 6, 7]. In this study, p-mTOR was almost exclusively detected in the cytoplasm in 63.2% of OL and 44.4% of OSCC cases, being absent in oral NM. These results indicate that mTOR pathway activation occurs in early stages of oral carcinogenesis. On the contrary, only a minority of OLP cases (10.3%) were positive for p-mTOR. In a previous study by Clark et al. [8], p-mTOR immunohistochemical expression was 81.9% sensitive and 100% specific in differentiating head and neck SCC from noncancerous oral mucosa, with the latter being uniformly negative. It is tempting thus to speculate that p-mTOR-positive OLP cases may have an increased potential for malignant transformation compared to p-mTOR-negative cases. If this hypothesis holds true, p-mTOR detection (which may be at least partially associated with cytoplasmic p-Akt positivity) could serve as a marker of increased risk of malignant progression in OLP.

Ribosomal protein S6 (pS6) is one major downstream target and effector of the mTOR pathway. Following activation by the ribosomal protein S6 kinase, phosphorylated pS6 participates in the regulation of cell proliferation, cell growth, and protein synthesis [23]. High expression of activated pS6 has been detected in a number of human cancers suggesting its possible usefulness as a cancer biomarker [12, 24]. Similarly, oral, head, and neck squamous cell carcinoma has demonstrated relatively high phosphorylated pS6 levels [8, 25]. Amornphimoltham et al. [25] have shown that phosphorylated pS6 is expressed at low levels in normal oral mucosa compared to oral dysplasia and SCC, supporting the notion that pS6 activation may represent an early event in the oral carcinogenesis process. In a recent study, Chaisuparat et al. [24] investigated the phosphorylation levels of ribosomal protein S6 in normal oral mucosa, oral epithelial dysplasia, and OSCC cases. Similar to our study, phosphorylated pS6 was detected in the majority of cases studied (including 50% of control normal mucosa samples, 100% of oral epithelial dysplasias, and 88.68% of OSCC). Oral dysplasias and OSCC showed a higher frequency of pS6 phosphorylation compared to normal mucosa. These authors also concluded that pS6 activation represents an early event in oral carcinogenesis and may serve as a useful diagnostic biomarker. 

In agreement with the aforementioned studies, our results confirm high activation levels of pS6 in oral precancerous and cancerous lesions. On the other hand, the vast majority of OLP cases showed phospho-pS6 positivity, albeit at significantly lower positivity and intensity levels compared to OL and OSCC; in fact, phospho-pS6 levels did not differ significantly between OLP and NM. Considering that pS6 phosphorylation is mediated by activated mTOR, which, in turn, is to a large extent controlled by Akt activation, our results collectively implicate that Akt/mTOR/pS6 pathway is active in the majority of oral premalignant and malignant lesions. In contrast, OLP cases, as a group, do not seem to be characterized by aberrant activation of the oncogenic Akt/mTOR/pS6 pathway. Nonetheless, the demonstrated variability at phosphorylated protein levels among OLP cases may indicate that a minority of OLPs do possess exuberant Akt/mTOR/pS6 signaling activity (manifested by positivity for p-Akt and p-mTOR and higher levels of phosphor-pS6), which could contribute to their premalignant potential. 

It should be emphasized that this is the first attempt to assess the actual activation status and role of Akt/mTOR/pS6 signaling in OLP. Using a bioinformatics model based on the “leader gene approach,” Giacomelli et al. [26] have suggested that PI3K signaling events mediated by Akt may play a role interrelating OLP and OSCC. 

In conclusion, the present study revealed that activation of the Akt/mTOR/pS6 signaling is a common event in oral premalignant (OL) and malignant (OSCC) lesions but occurs only in a subset of OLP cases. It is interesting to note that differences in the localization of p-Akt in the nuclear and cytoplasmic compartments may account for a variable activation status, a notion that needs further investigation. In addition, investigating the expression and activation levels of other molecules involved in the complex mTOR signaling may provide more information on the actual activation status of this pathway in oral carcinogenesis. Although no follow-up data were available to record the malignant transformation rate of OLP cases studied, it is conceivable that lesions harboring aberrations in the Akt/mTOR/pS6 signaling may bear a closer molecular similarity to actual premalignant lesions and may be at increased risk for cancer development. Furthermore, the advent of molecular targeted therapies against Akt and mTOR holds promise for their use in the context of premalignant disease, which may encompass selected cases of OLP. Confirmation of these hypotheses will necessitate a large prospective study of OLP cases with careful recording of the clinical features and long follow-up. It will be interesting to correlate the presence and frequency of molecular aberrations involving the Akt/mTOR/pS6 or other related pathways with specific OLP clinical subtypes, given that specific clinical forms of the disease (such as erosive, atrophic, and/or plaque-like OLP) have been associated with a higher malignant transformation rate [1, 2]. Moreover, long-term follow-up will reveal those cases demonstrating malignant transformation (or even the development of a preceding stage of histologic features of dysplasia) allowing the determination of the actual prognostic significance and clinical value of specific molecular markers.

## Figures and Tables

**Table 1:** Percentage of positive cases and average positivity, intensity, and total scores for nuclear p-Akt per lesion category.

	Number and % of positive cases	Average positivity score	Average intensity score	Average total score
OLP	37/40 (92.5%)	2.00	1.53	3.53
NM	9/9 (100%)	1.78	1.78	3.56
OL	17/17 (100%)	2.94*	2.71*	5.65*
OSCC	7/10 (70%)	1.80	1.30	3.10

OLP: oral lichen planus; NM: normal mucosa; OL: oral leukoplakia; OSCC: oral squamous cell carcinoma.

*Statistical significant differences (*P* < 0.05), compared to OLP.

**Table 2:** Percentage of positive cases and average positivity, intensity, and total scores for cytoplasmic p-Akt per lesion category.

	Number and % of positive cases	Average positivity score	Average intensity score	Average total score
OLP	2/40 (5%)	0.05	0.08	0.05
NM	0/9 (0%)	0	0	0
OL	11/17 (64.7%)	0.94*	1.06*	2.00*
OSCC	7/10 (70%)	1.10*	1.10*	2.20*

OLP: oral lichen planus; NM: normal mucosa; OL: oral leukoplakia; OSCC: oral squamous cell carcinoma.

*Statistical significant differences (*P* < 0.05), compared to OLP.

**Table 3:** Percentage of positive cases and average positivity, intensity, and total scores for p-mTOR per lesion category.

	Number and % of positive cases	Average positivity score	Average intensity score	Average total score
OLP	4/39 (10.3%)	0.154	0.154	0.31
NM	0/10 (0%)	0	0	0
OL	12/19 (63.2%)	1.00*	1.05*	2.05*
OSCC	4/9 (44.4%)	0.78*	0.56*	1.33*

OLP: oral lichen planus; NM: normal mucosa; OL: oral leukoplakia; OSCC: oral squamous cell carcinoma.

*Statistical significant differences (*P* < 0.05), compared to OLP.

**Table 4:** Percentage of positive cases and average positivity, intensity, and total scores for phospho-pS6 per lesion category.

	Number and % of positive cases	Average positivity score	Average intensity score	Average total score
OLP	36/40 (90%)	1.28	1.28	2.52
NM	9/9 (100%)	1.78	1.78	3.56
OL	16/16 (100%)	2.25*	1.81	4.06*
OSCC	8/9 (88.9%)	2.33*	2.33*	4.67*

OLP: oral lichen planus; NM: normal mucosa; OL: oral leukoplakia; OSCC: oral squamous cell carcinoma.

*Statistical significant differences (*P* < 0.05), compared to OLP.
